# Genome-wide analysis of the *HSP20* gene family and its response to heat and drought stress in Coix (*Coix lacryma-jobi* L.)

**DOI:** 10.1186/s12864-023-09580-2

**Published:** 2023-08-24

**Authors:** Yangguang Hua, Qiao Liu, Yufeng Zhai, Limin Zhao, Jinjian Zhu, Xiaodan Zhang, Qiaojun Jia, Zongsuo Liang, Dekai Wang

**Affiliations:** 1 Key Laboratory of Plant Secondary Metabolism Regulation in Zhejiang Province, College of Life Sciences and Medicine, Zhejiang Sci-Tech University, Hangzhou, 310018 Zhejiang People’s Republic of China; 2 Jinyun County Agriculture and Rural Bureau, Jinhua, 321400 People’s Republic of China; 3 State Key Laboratory of Dao-Di Herbs, 100700, Beijng, People’s Republic of China

**Keywords:** Medicine and food homology plant, Coix, Heat shock protein 20, Gene family, Abiotic stress

## Abstract

**Background:**

Heat shock protein 20 (HSP20) is a member of the heat stress-related protein family, which plays critical roles in plant growth, development, and response to abiotic stresses. Although many *HSP20* genes have been associated with heat stress in numerous types of plants, little is known about the details of the *HSP20* gene family in Coix. To investigate the mechanisms of the *ClHSP20* response to heat and drought stresses, the *ClHSP20* gene family in Coix was identified and characterized based on genome-wide analysis.

**Results:**

A total of 32 putative *ClHSP20* genes were identified and characterized in Coix. Phylogenetic analysis indicated that ClHSP20s were grouped into 11 subfamilies. The duplicated event analysis demonstrated that tandem duplication and segment duplication events played crucial roles in promoting the expansion of the *ClHSP20* gene family. Synteny analysis showed that Coix shared the highest homology in 36 *HSP20* gene pairs with wheat, followed by 22, 19, 15, and 15 homologous gene pairs with maize, sorghum, barley, and rice, respectively. The expression profile analysis showed that almost all *ClHSP20* genes had different expression levels in at least one tissue. Furthermore, 22 of the 32 *ClHSP20* genes responded to heat stress, with 11 *ClHSP20* genes being significantly upregulated and 11 *ClHSP20* genes being significantly downregulated. Furthermore, 13 of the 32 *ClHSP20* genes responded to drought stress, with 6 *ClHSP20* genes being significantly upregulated and 5 *ClHSP20* genes being significantly downregulated.

**Conclusions:**

Thirty-two *ClHSP20* genes were identified and characterized in the genome of Coix. Tandem and segmental duplication were identified as having caused the expansion of the *ClHSP20* gene family. The expression patterns of the *ClHSP20* genes suggested that they play a critical role in growth, development, and response to heat and drought stress. The current study provides a theoretical basis for further research on *ClHSP20s* and will facilitate the functional characterization of *ClHSP20* genes.

**Supplementary Information:**

The online version contains supplementary material available at 10.1186/s12864-023-09580-2.

## Introduction

To survive and adapt to the adversity of high temperatures, plants developed complex self-defence mechanisms over the course of evolution. Heat shock proteins (HSPs), important proteins synthesized at high temperatures, have been shown to participate in various responses to environmental stress and regulate many developmental processes [1]. HSPs can generally be classified into five families, including HSP100, HSP90, HSP70, HSP60, and HSP20, based on their molecular weight [1, 2]. As an important group of molecular chaperones, HSPs are widely found in living organisms from prokaryotic to eukaryotic organisms [2–4]. HSP20 is also called a small heat shock protein (sHsp) because its molecular weight ranges from 15 to 42 kDa [5]. Studies have shown that HSP20 is the largest HSP family in plants, and the HSP20 protein is the most abundant protein induced by elevated temperature-associated stress in many higher plants [6, 7]. To accommodate various stresses, the structure of HSP20s has developed significant diversity but members share a highly conservative α-crystalline domain (ACD) that allows them to be recognized [6]. HSP20s can also act as ATP-independent molecular chaperones to prevent protein denaturation in their substrates by forming oligomeric protein complexes, thus promoting the adaptability of plants to external environmental stress [8–10].

Numerous investigations have revealed that most *HSP20s* are strongly induced by abiotic and biotic stresses such as heat, drought, salinity, cold, heavy metals, anoxia, and some pathogens, thus enhancing the tolerance of plants to these stresses [11–13]. Many studies have shown that *HSP20* genes play a critical role in abiotic stress in plants. For example, Arabidopsis *HSP20* overexpression was found to induce high levels of antioxidant enzyme expression, resulting in enhanced tolerance to high temperature, salinity, osmotic, and oxidative stresses [14]. Overexpression of rice *OsHsp17.0* and *OsHsp23.7* was shown to increase tolerance of both drought and salt [15]. In tomato, the overexpression of *SlHSP17.7* increases the tolerance of plants to cold stress and reduces the accumulation of reactive oxygen species (ROS) [16]. Overexpression of *PpHSP20-32* in peach increases plant height and enhances improved thermotolerance [17].

Coix (*Coix lacryma-jobi* L), also called adlay or Job’s tears, is a medicinal and food-homogeneous cereal crop. It is widely planted throughout the world and is mainly planted in the countries of Northeast Asia, such as China, Korea, and Japan [18]. Coix is praised as the “king of cereals”, as its seed has the highest protein content among cereal crops and contains more than 30 nutritional and functional ingredients. As its pharmacological activities are derived from its oil components, Coix seed has long been used as a traditional Chinese medicine to promote urination, serve as a diuretic, improve immunity, etc. [19]. Since the seeds are rich in nutrients and medicinal ingredients, Coix is considered a homologous substance of medicine and food in China [19]. Furthermore, Coix plants have good adaptability to many biotic and abiotic stresses, including drought, waterlogging, low pH, and diseases [20].

Since the discovery of the importance of *HSP20* genes in response to various biotic and abiotic stresses, the *HSP20* family of genes has been identified and characterized in dozens of plant species, such as Arabidopsis (*Arabidopsis thaliana*) [21], rice (*Oryza sativa*) [7], soybean (*Glycine max*) [22], tomato (*Solanum lycopersicum*) [23], cotton (*Gossypium hirsutum*) [24], potato (*Solanum tuberosum*) [25], grape (*Vitis vinifera*) [26], barley (*Hordeum vulgare*) [27], dove tree (*Davidia involucrata*) [28], maize (*Zea mays*) [29], rowan (*Sorbus pohuashanensis*) [30], and pepper (*Capsicum annuum*) [31], using genome-wide functional analysis. However, the *HSP20* genes have not been thoroughly identified in Coix. In this study, a comprehensive and systematic analysis of the *HSP20* gene family in Coix was carried out with the aim of (i) identifying members of the *ClHSP20* gene family, the chromosome position of the *ClHSP20* gene, and the conserved domain in ClHSP20; (ii) classifying these members based on phylogenetic analyses; (iii) identifying gene duplication in *ClHSP20s*; and (iv) exploring the gene expression patterns in Coix tissues and response to heat and drought responses.

## Results

### Identification and sequence analysis of *ClHSP20* genes in Coix

A total of the 32 *ClHSP20* genes were identified in the Coix genome based on BLASTp and HMMER searches and confirmed by the CDD, Pfam, and SMART databases. The length of the ClHSP20 proteins ranged from 114 aa (ClHSP20-29) to 496 aa (ClHSP20-3), and the corresponding molecular weight (MW) ranged from 13.07 to 54.10 kDa. Furthermore, the predicted isoelectric point (pI) of ClHSP20 proteins ranged between 4.66 (ClHSP20-21) and 10.44 (ClHSP20-4), and most of these values were greater than 7.0, suggesting that they encode alkaline proteins. The prediction of subcellular localization showed that 27 of the 32 ClHSP20 proteins were localized to the cytoplasm or nucleus; 2 were localized in the plastid; three were localized in the mitochondria; and one was localized in the peroxisome (Table 1).

**Table 1:** Summary of characteristics of *ClHSP20* gene family members in Coix genome

Named	Chromosome	Sequence ID	Position	Strand	Amino acid	MW (kD)	pI	Subcellular localization
ClHSP20-1	chr1	Cl017461_T1	7,430,513–7431364	-	170	18.25	6.66	Cytoplasmic
ClHSP20-2	chr1	Cl017649_T1	9,891,060–9892182	-	220	23.92	6.06	Mitochondrial
ClHSP20-3	chr1	Cl018009_T1	16,450,885–16455531	+	496	54.10	4.68	Nuclear
ClHSP20-4	chr1	Cl020770_T2	171,626,360–171631383	+	337	37.73	10.44	Mitochondrial
ClHSP20-5	chr1	Cl021459_T1	184,742,686–184,743,813	-	210	22.42	5.77	Cytoplasmic
ClHSP20-6	chr2	Cl001127_T1	16,020,200–16021333	-	246	26.96	8.73	Chloroplast
ClHSP20-7	chr2	Cl001129_T1	16,151,000–16151479	+	159	17.93	7.99	Cytoplasmic
ClHSP20-8	chr2	Cl001241_T1	17,579,510–17579989	+	159	17.93	7.99	Cytoplasmic
ClHSP20-9	chr2	Cl003175_T1	80,135,935–80136895	-	184	20.00	9.43	Chloroplast
ClHSP20-10	chr2	Cl003267_T1	92,430,345–92431432	-	247	26.67	9.44	Chloroplast
ClHSP20-11	chr2	Cl003268_T1	92,774,087–92775087	-	213	23.06	9.17	Chloroplast
ClHSP20-12	chr2	Cl003628_T1	126,547,533–126,548,688	+	229	25.22	9.3	Cytoplasmic
ClHSP20-13	chr2	Cl003629_T1	126,578,105–126579711	+	171	18.67	7.06	Cytoplasmic
ClHSP20-14	chr2	Cl004747_T1	158,600,368–158601441	-	279	30.77	5.65	Cytoplasmic
ClHSP20-15	chr2	Cl004749_T1	158,605,023–158606123	-	346	37.73	8.72	Cytoplasmic
ClHSP20-16	chr3	Cl027824_T1	148,251,284–148,251,733	+	149	15.95	9.05	Peroxisome
ClHSP20-17	chr3	Cl028061_T2	155,833,776–155,834,935	+	265	29.11	6.47	Mitochondrial
ClHSP20-18	chr4	Cl007630_T1	17,625,897–17,626,860	-	154	17.23	7.11	Nuclear
ClHSP20-19	chr4	Cl009706_T1	89,406,095–89407055	+	184	20.00	9.43	Chloroplast
ClHSP20-20	chr5	Cl029676_T1	7,111,334–7,113,134	+	199	21.94	4.83	Cytoplasmic
ClHSP20-21	chr5	Cl029815_T3	10,049,528–10055486	+	451	48.89	4.66	Cytoplasmic
ClHSP20-22	chr6	Cl014691_T1	49,367,161–49,368,437	+	287	30.97	8.48	Cytoplasmic
ClHSP20-23	chr6	Cl014692_T1	49,380,046–49380624	+	138	14.95	10.41	Nuclear
ClHSP20-24	chr6	Cl014693_T1	49,383,474–49,384,315	+	169	17.71	7.73	Cytoplasmic
ClHSP20-25	chr6	Cl016300_T1	135,636,265–135,636,729	-	154	17.16	5.55	Cytoplasmic
ClHSP20-26	chr6	Cl016301_T1	135,655,061–135655519	+	152	17.14	6.85	Cytoplasmic
ClHSP20-27	chr6	Cl016303_T1	135,746,040–135746510	+	156	17.63	5.56	Cytoplasmic
ClHSP20-28	chr6	Cl016305_T1	135,770,013–135770981	-	213	23.72	9.51	Cytoplasmic
ClHSP20-29	chr8	Cl035644_T1	2,837,455–2,838,751	+	114	13.07	9.67	Nuclear
ClHSP20-30	chr9	Cl023438_T1	24,045,016–24045492	-	158	18.05	7.99	Cytoplasmic
ClHSP20-31	chr9	Cl023439_T1	24,046,084–24046572	+	162	17.88	5.97	Cytoplasmic
ClHSP20-32	contig326	Cl041628_T1	1,273,067–1274577	+	347	36.81	9.41	Cytoplasmic

### Phylogenetic analysis and classification of *ClHSP20* genes

**Fig. 1:**
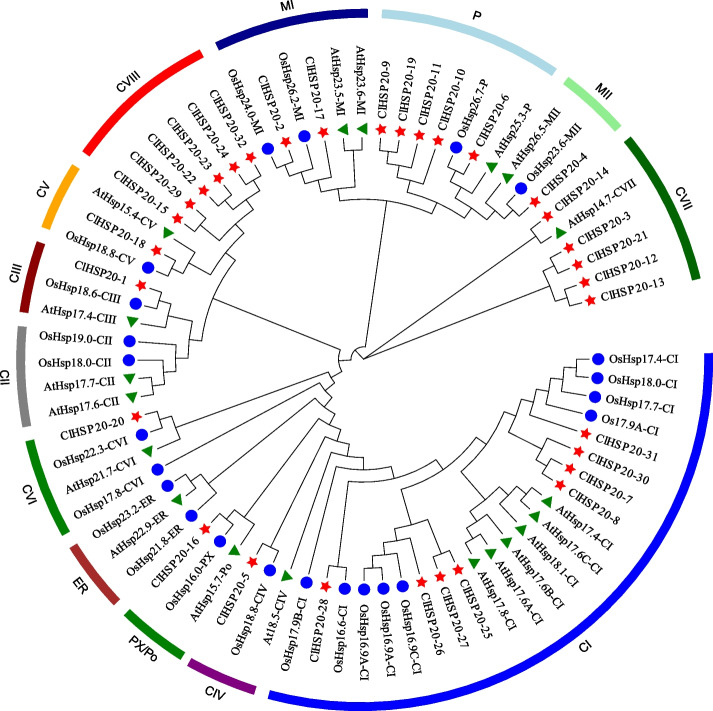
Phylogenetic relationship of HSP20 proteins in Arabidopsis (At), rice (Os), and Coix (Cl). Full-length HSP20 protein sequences were aligned using the Clustal X 1.83 software, and the neighbor-joining (NJ) phylogenetic tree was constructed using MEGA 7.0 with 1,000 bootstrap replicates. HSP20 in different subfamilies are labeled with different colors. The species names are abbreviated as follows: At, *Arabidopsis thaliana*; Os, *Oryza sativa*, and Cl, *Coix lacryma-jobi*. C, cytoplasmic/nuclear; ER, endoplasmic reticulum; P, plastid; Po, peroxisome; M, mitochondria

To better understand the evolutionary history and relationship of the *HSP20* gene family, an unrooted phylogenetic tree of neighbour-joining (NJ) was constructed using 73 HSP20 amino acid sequences from Arabidopsis (19), rice (22), and Coix (32). The selected HSP20s were divided into 13 subfamilies according to a previous classification (Fig. 1). Among these subfamilies, 32 ClHSP20 proteins were assigned to 10 identified subfamilies (CI, CIII, CV, CVI, CVII, CIV, PX/Po, P, MI, and MII) and a new subfamily, nucleocytoplasmic VIII (CVIII), which was identified in this study (Fig. 1). Among the 32 ClHSP20 proteins, most (23 of 32) were cytoplasmic (C) proteins (classified into CI–CVII); five were plastidic (P) proteins; three were mitochondrial (M) proteins (two subfamilies); and only one was a peroxisomal (Px) protein. However, Coix did not have HSP20 proteins located in the endoplasmic reticulum (ER) or CII subfamilies.

### Chromosomal distribution and gene duplication of *ClHSP20*

**Fig. 2:**
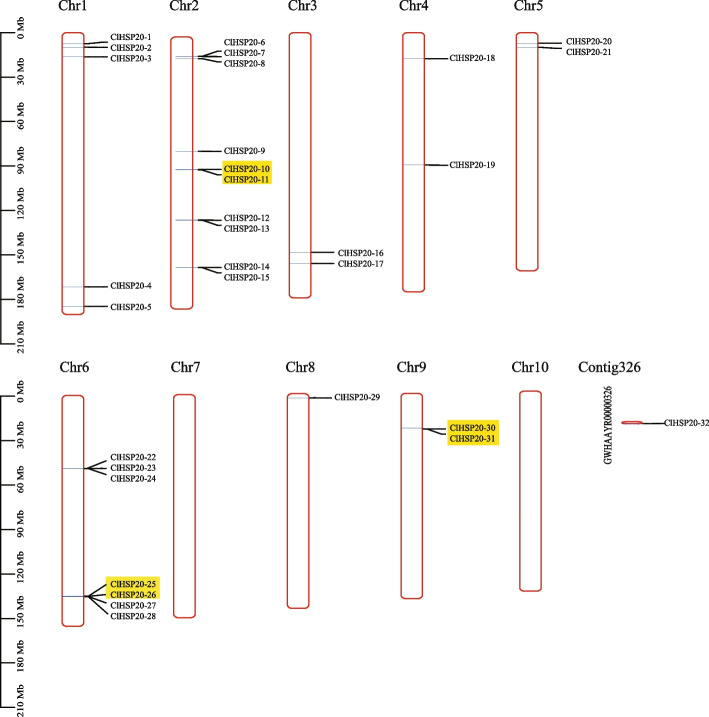
Chromosomal distributions of *ClHSP20* genes. The length of the chromosomes is represented by the scale on the left. Tandemly duplicated *ClHSP20s* are highlighted with yellow color

Based on gene coordinate annotation data, 31 of the 32 *ClHSP20* genes were unevenly distributed on 8 of the 10 chromosomes in the Coix genome; *ClHSP32* was the exception and was not assigned to any chromosomes (Fig. 2). Chr 2 had the highest number of *ClHSP20* genes (10), Chr 6 and 1 had seven and five *ClHSP20* genes, respectively. Chr 3, 4, and 9 all had two *ClHSP20* genes; Chr 8 has only one *ClHSP20* gene. Moreover, there were no *ClHSP20* genes on chr 7 and 10.

An analysis of duplication events showed that a total of three *ClHSP20* groups (*ClHSP20-10/ClHSP20-11; ClHSP20-25/ClHSP20-26; ClHSP20-30/ClHSP20-31*) were the result of tandem duplication events. The tandem duplicated genes were located on chrs 2, 6, and 9. Additionally, a total of three pairs of segmental duplication events were detected, including for *ClHSP20-2/ClHSP20-17, ClHSP20-14/ClHSP20-29*, and *ClHSP20-22/ClHSP20-32* (Fig. 2; Table 2). In detail, 1, 2, 3, 6, 8, and contig_326 contained one segmental duplication gene pair. The results demonstrated that tandem and segmental duplication promoted the expansion of the *ClHSP20* gene family.

**Fig. 3:**
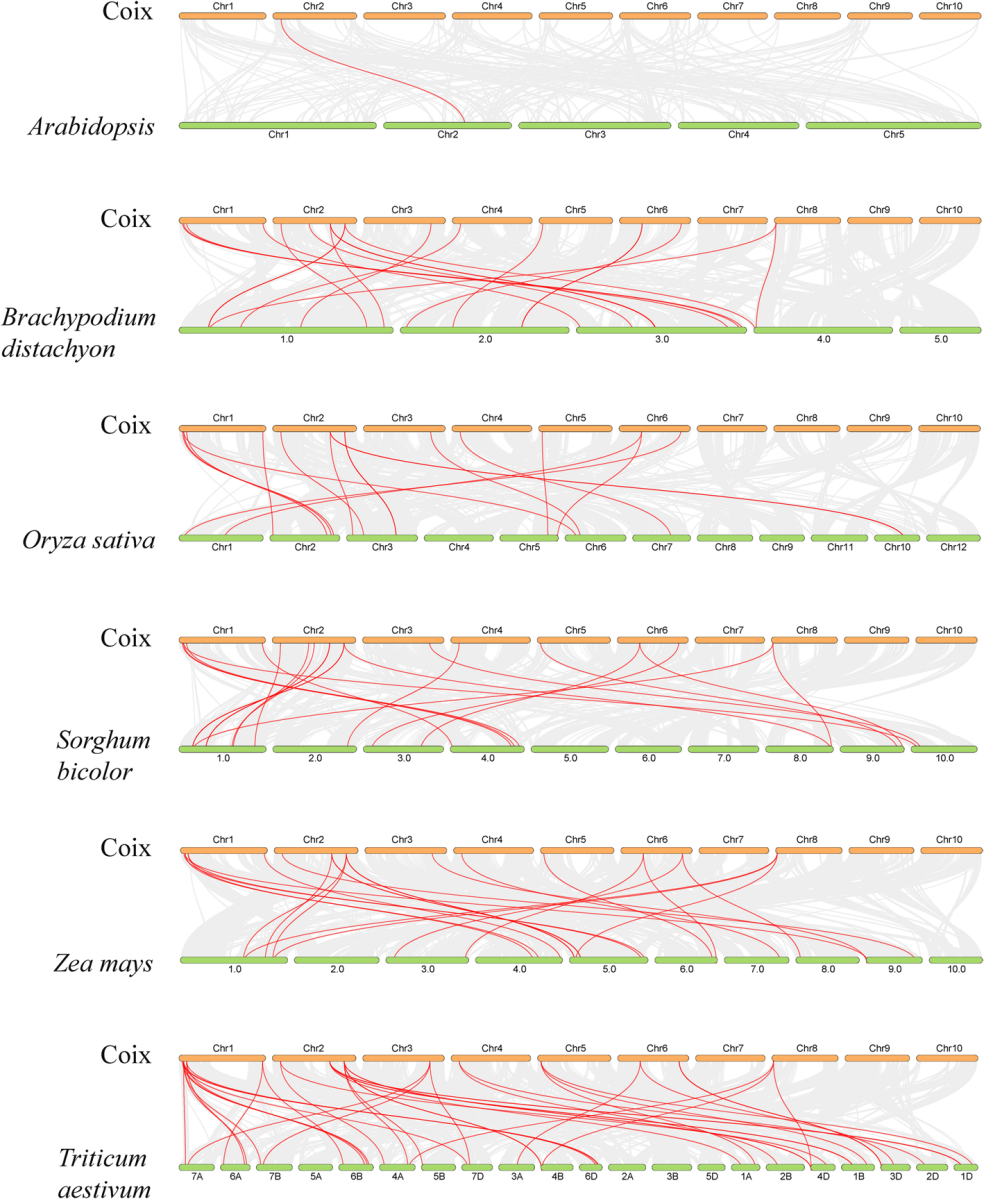
Synteny analysis of *HSP20* genes between Coix and other species. The gray lines represent the collinear blocks within the genome, and the red lines highlight the syntenic pairs of *HSP20* genes. The number of chromosomes is displayed in the middle of each chromosome.

An analysis was conducted to further investigate the phylogenetic relationship, potential evolutionary link and collinearity of members of the *HSP20* gene family between Coix and other species, including Arabidopsis, rice (*Oryza sativa*, MSUv7.0), maize (*Zea mays*, ZmB73_4a.53), sorghum (*Sorghum bicolor*, Sbicolor_79), brachypodium (*Brachypodium distachyon*, v3.0.52), barley (*Hordeum vulgare*, v3), millet (*Setaria italica*, v2.0), and wheat (*Triticum aestivum*, IWGSC.53). The collinearity relationship among HSP20 genes in different species is shown in Fig. 3. The *ClHSP20* genes had different degrees of collinear relationships with other species. The most orthologous *HSP20* pairs (36) were detected between Coix and wheat, followed by maize, sorghum, barley, and rice, with 22, 19, 15, and 15 homologous pairs with Coix, respectively. However, there was only one homologous pair between Coix and Arabidopsis (Fig. 3), which demonstrated that Coix is more closely related to cereal plants than to Arabidopsis.

To investigate the detailed evolutionary information of the *HSP20* gene family, the Ka/Ks ratios of the *ClHSP20* gene pairs were calculated. The results showed that the Ka/Ks values of six duplicated *ClHSP0* gene pairs, excluding *ClHSP20-14/ClHSP20-29*, were lower than 1, suggesting that these *ClHSP20* genes had undergone purification selective pressure during their evolution (Table 2). The Ka/Ks ratio of the other duplicated gene pairs (*ClHSP20-14/ClHSP20-29*) was greater than 1 (1.116), indicating that these two *ClHSP20* genes had undergone positive selection. The divergence times of the duplicated gene pairs ranged from 19.38 to 62.73 million years ago (Mya) and averaged 44.46 Mya. The three segmental duplication events occurred in the same era (averaged 48.76 Mya) (Table 2). In particular, the divergence times of one tandem duplication gene pair (*ClHSP20-10/ClHSP20-11*) (19.38 Mya) were much earlier than the occurrence of segmental duplication (averaged 48.76 Mya), while two tandem duplication gene pairs (*ClHSP20-25/ClHSP20-26 and ClHSP20-30/ClHSP20-31*) were later than the occurrence of segmental duplication.

### Gene structure and protein structure of *ClHSP20* genes

**Fig. 4:**
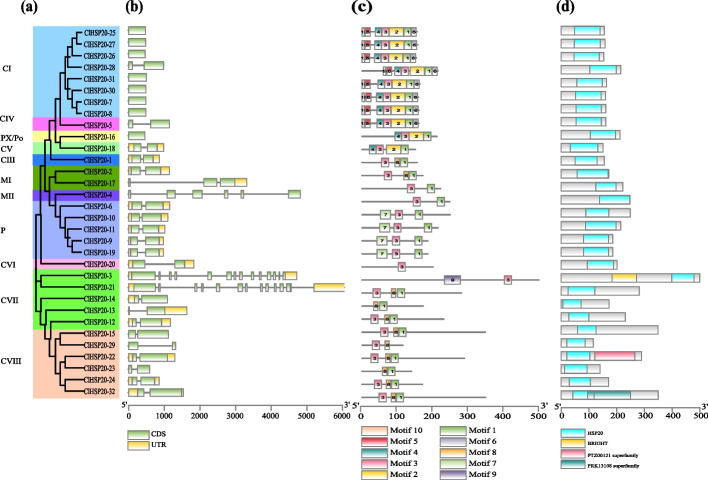
Phylogenetic relationships, conserved protein motifs, CCD domain, and gene structures of the ClHSP20s. **a**. The NJ phylogenetic tree of ClHSP20 was constructed with MEGA 7.0. **b** The Exon–intron structure of *ClHSP20* genes. The untranslated regions, exons, and introns are indicated by yellow boxes, green boxes, and solid gray lines, respectively. The scale at the bottom is in bp; **c** Conserved motifs in ClHSP20 proteins. Boxes of different colors represent different motifs; **d** Conserved domain of ClHSP20. The distinct colored boxes represent different conserved domains

The structure of the *ClHSP20* genes was determined by aligning genomic DNA with full-length *ClHSP20* cDNA. As shown in Fig. 4, most of the *ClHSP20* genes (20, accounting for 62.5%) had one intron, eight (accounting for 25.0%) were intronless. The rest of 4 *ClHSP20* genes, including *ClHSP20-17* (2 introns), *ClHSP20-4* (5 introns), *ClHSP20-3* (13 introns), and *ClHSP20-21* (12 introns), had two or more introns (Fig. 4). In particular, all the tandemly duplicated *ClHSP20* genes were intronless, while the pair of three segmentally duplicated *ClHSP20* genes had two introns (Fig. 4b).

The conserved motifs of the ClHSP20 protein sequences were analysed using the MEME online tool, and 10 motifs were detected. Motif 1 and motif 2 were found in approximately all ClHSP20 proteins (Fig. 4c). The details of the motif pattern and structure were used to support the information about the ClHSP20 phylogenetic relationship.

Conserved domain analysis showed that all of the ClHSP20 proteins harboured a highly conserved ACD domain (HSP20). In addition, five ClHSP20s contained additional domains: ClHSP20-22 contained a PTZ00121 superfamily domain, ClHSP20-32 contained a PRK13108 superfamily domain, ClHSP20-4 contained a DUF4050 domain, and ClHSP20-3 and ClHSP20-21 contained a BRIGHT domain (Fig. 4d). In conclusion, the position and number of exons and introns, as well as the motif pattern and conserved domain, supported the phylogenetic relationship of ClHSP20s.

### GO term, KEGG pathway enrichment and analysis of the PPI network of *ClHSP20* genes

**Fig. 5:**
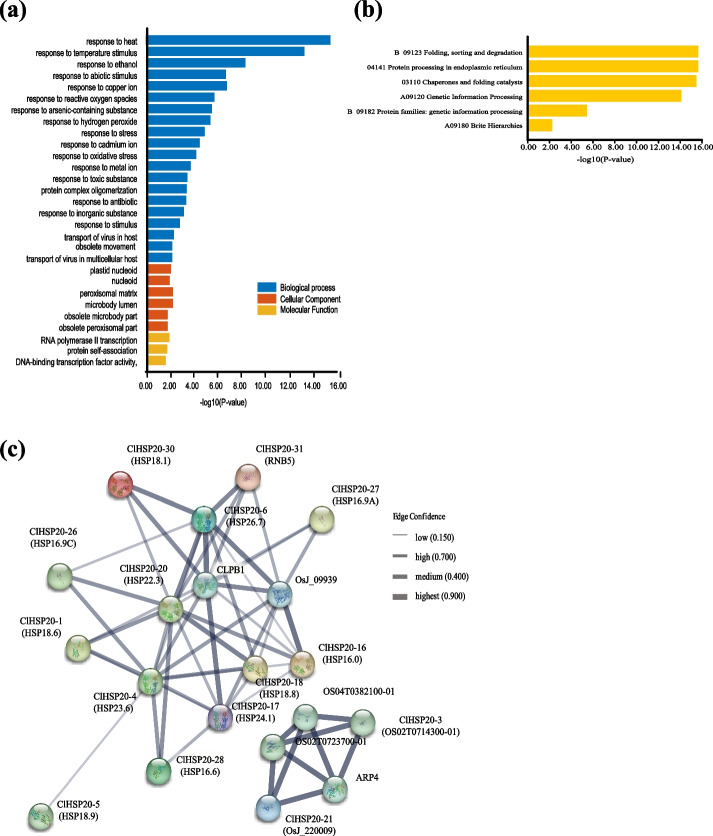
Gene ontology (GO), KEGG enrichment analysis, and predicted protein–protein interaction (PPI) networks of ClHSP20s. **a** GO enrichment analysis of ClHSP20s. The X and Y axes represent the -log10 (*P* value) and the information on GO terms, respectively. **b** KEGG enrichment analysis of ClHSP20s. The X and Y axes represent the -log10 (*P* value) and the information on the KEGG pathway, respectively. **C** PPI network of significant *ClHSP20* genes in Coix. Nodes represent proteins and gray lines indicate interactions between nodes. Different thicknesses of grey edges indicated the degree of protein–protein associations

To further elucidate the functions of the *ClHSP20* genes, GO and KEGG enrichment analyses were performed (Fig. 5). The three main GO categories that were enriched in ClHSP20 proteins included 20 biological process terms, 6 cellular component terms, and 3 molecular function terms (Supplementary Table S1). For the biological process GO terms, the highly enriched terms included response to heat (GO:0009408), which contained ClHSP20-1, ClHSP20-2, ClHSP20-6, ClHSP20-7, ClHSP20-8, ClHSP20-16, ClHSP20-17, ClHSP20-18, ClHSP20-25, ClHSP20-27, ClHSP20-28, and ClHSP20-30; response to temperature stimulus (GO:0009266); response to ethanol (GO:0045471); response to abiotic stimulus (GO:0009628), etc. In terms of cellular components, ClHSP20 proteins were components of the terms plastid nucleoid (GO:0042646); nucleoid (GO:0009295); peroxisomal matrix (GO:0005782); microbody lumen (GO:0031907), etc. In terms of molecular function, the ClHSP20 proteins were classified as exhibiting sequence-specific DNA binding of the RNA polymerase II transcription regulatory region (GO:0000977), protein self-association (GO:0043621), DNA binding transcription factor activity, and RNA polymerase II specificity (GO:0000981) (Fig. 5a). Furthermore, the KEGG pathway enrichment study identified six pathways involved in the different functions of the *ClHSP20* genes. The highly enriched pathways included folding, sorting, and degradation (B09123); protein processing in the endoplasmic reticulum (04141); chaperones and folding catalysts (03110); genetic information processing (A09120); protein families: genetic information processing (B09182); and brite Hierarchies (A09180) (Fig. 5b; Supplementary Table S2).

To determine interactions among ClHSP20s and related proteins, a PPI interaction network was constructed using the STRING database based on rice protein orthologues. A total of the 32 ClHSP20 proteins had rice orthologues with identities ranging from 36.5 to 88.8%. As shown in Fig. 5c, the PPI network consisted of 20 nodes and 54 edges, suggesting that these ClHSP20 proteins interacted with each other and with other proteins and participated in some biological processes. For example, ClHSP20-20 shared a high degree of interaction with other ClHSP20 members, which was associated with 10 ClHSP20 proteins and the CLPB1 protein. ClHSP20-21 is associated with ARP4, which is involved in several developmental processes, including the organization of plant organs, flowering time, other development, flower senescence, and fertility. The results of the GO, KEGG, and PPI analyses demonstrated that *ClHSP20* genes were related to the response, development, and protein modification processes that occur to respond to abiotic stresses.

### Prediction of the motif in the *ClHSP20* gene promoter

**Fig. 6:**
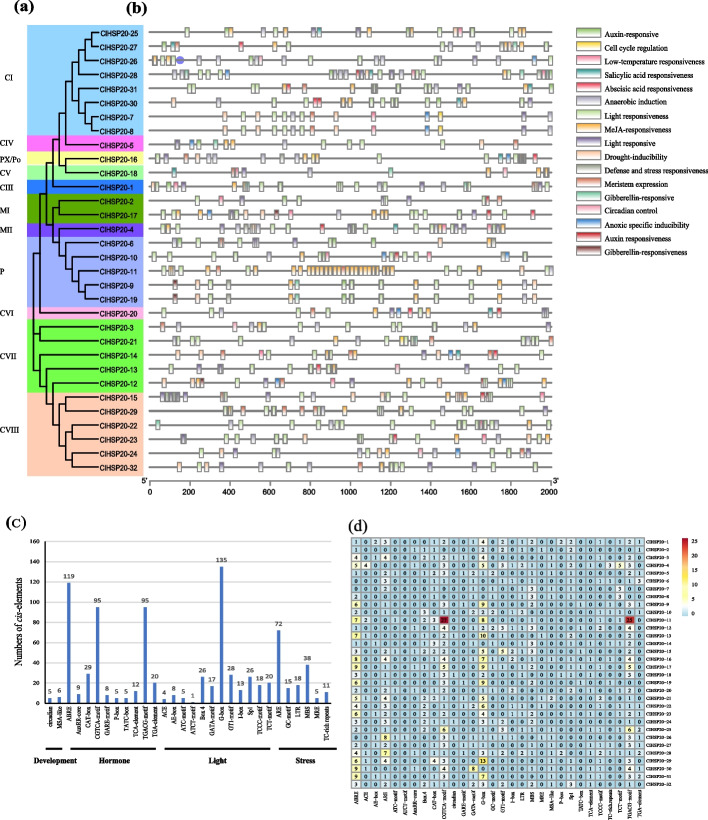
Diagram of Cis-element in the promoter region of *ClHSP20* genes. The black line represents the length of the *ClSHP20* gene promoter. Rectangular boxes of distinct colored boxes represent the different types of cis-acting elements

To investigate the potential physiological functions of *ClHSP20*, the upstream 2,000 bp of the starting site of the *ClHSP20* genes was selected and analysed with the PlantCARE online database. A total of 868 *cis*-acting elements involved in hormone response, plant growth and development, and abiotic and biotic stress responses were found to be present in the *ClHSP20* promoter. The distribution of the *cis*-acting elements was schematically depicted (Fig. 6b; Supplementary Table S3). The elements of the promoter of the *ClHSP20* genes in tandem and segmental duplication were generally similar (Fig. 6a, and b). There were two categories of *cis*-elements related to development, 10 to the hormone response, 12 to the light response, and 6 to the stress response. Among these *cis*-elements, the G box (135 in total) involved in the light response accounted for the largest category, followed by the ABREs (11 in total) involved in the abscisic acid response, which accounted for the second largest category. Interestingly, the promoter of *ClHSP20-11* contained many CGTCA motifs (27 in total) and TGACG motifs (25 in total) involved in the MeJA response, indicating that the *ClHSP20-11* functions were probably related to the damage response. There were many types of elements in the stress response, such as anaerobic induction (ARE motif: AAACCA) (72 in total), drought inducibility (MBS motif: CAACTG) (38 in total), low-temperature response (LTR motif: CCGAAA) (18 in total), and anoxic (GC-motif: CCCCCG) (15 in total) (Figs. 6c, d; Supplementary Table S3). All *ClHSP20s* possessed at least one *cis*-element associated with the stress response, hinting that the functions of *ClHSP20s* were associated with the response to abiotic stress.

### Profiling of *ClHSP20* gene expression in different tissues

**Fig. 7:**
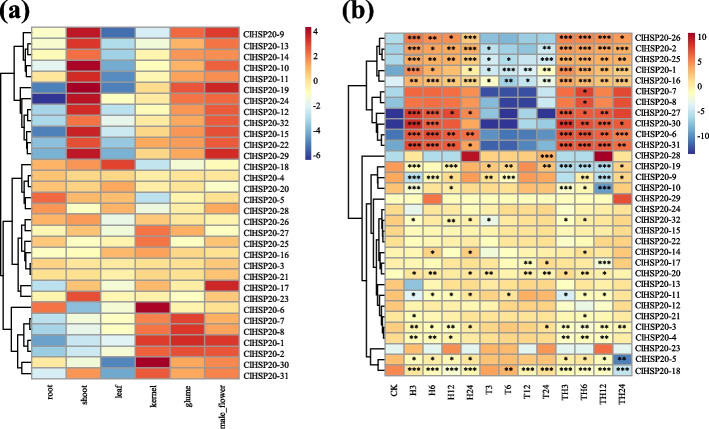
Expression pattern of *ClHSP20* genes in different tissues and under heat and drought treatment. **a** Expression pattern of *ClHSP20* genes in different tissues. The heatmap was generated based on public RNA-seq data (BioProject number: PRJNA544168). **b** Expression pattern of *ClHSP20* genes under heat and drought treatment. The heatmap was generated based on public RNA-seq data (BioProject number: PRJNA812268). The red and blue color scale represents log2 normalized values of TPM, and indicates a relative expression level. T, Heat treatment (40 °C) for 0 h, 3 h, 6 h, 12 h, and 24 h, respectively; H, drought treatment with 15% PEG6000 for 0 h, 3 h, 6 h, 12 h and 24 h, respectively; TH, Heat and drought treatment with 40℃ + 15% PEG6000 for 0 h, 3 h, 6 h, 12 h and 24 h, respectively. The TPM values of three biological duplications were subjected to an analysis of variance (ANOVA), and a comparison of means was carried out by Student’s t-test. ^***^ *P* < 0.001, ^**^ *P* < 0.01, ^*^P < 0.05

The TPM (transcripts per kilogram base per million mapped reads) values of the 32 *ClHSP20* genes were acquired from the transcriptome data for six distinct tissue samples (root, shoot, leaf, kernel, glume, and male flower) based on the Coix RNA-seq data (PRJNA544168). To investigate the expression of *ClHSP20* transcripts, a heatmap was generated with the corresponding log2-TPM values of the six organs using the heatmap tool (Fig. 7a; Supplementary Table S4). Almost all *ClHSP20* genes were detected to have different degrees of expression levels in at least one tissue. Some *ClHSP20* genes showed a differential expression pattern; for example, *ClHSP20-9*, *ClHSP20-13*, *ClHSP20-14*, *ClHSP20-10*, and *ClHSP20-9* were expressed at the highest levels in the stem, followed by the male flower, glume, kernel, and root, but could hardly be detected in the leaf. *ClHSP20-19*, *ClHSP20-12*, *ClHSP20-32*, *ClHSP20-15*, *ClHSP20-22*, and *ClHSP20-29* also showed similar expression patterns and were expressed at the highest levels in shoots, followed by male flowers, glumes, and kernels, but could hardly be detected in leaves and roots. Several *ClHSP20* genes showed tissue-specific expression patterns. For example, *ClHSP20-6*, *ClHSP20-7*, *ClHSP20-8*, *ClHSP20-1*, *ClHSP20-2*, *ClHSP20-30*, and *ClHSP20-31* were expressed preferentially in reproductive organs (kernel, glume and male flower), while their expression was lower in vegetative tissues (root, shoot, and leaf). Some *ClHSP20* genes were expressed in all tested tissues but were highly expressed in certain organs. For example, *ClHSP20-18* was most highly expressed in the leaf, *ClHSP20-5* in the root, *ClHSP20-17* in the male flower, *ClHSP20-23* in the shoot, and *ClHSP20-6* and *ClHSP20-30* in the kernel.

### Analysis of *ClHSP20* gene expression in response to heat and drought stress

An analysis of the *cis*-acting elements showed that most of the promoters of *ClHSP20* genes harboured stress response elements. To investigate the potential role of *ClHSP20* genes under heat and drought stress, the TPM values of the 32 *ClHSP20* genes were acquired from the transcriptome data based on the Coix RNA-seq data (PRJNA812268). The results showed that 22 out of the 32 *ClHSP20* genes had a significant (*P* < 0.05) response to heat stress at some or all treatment time steps (3 h, 6 h, 12 h and 24 h), of which 11 were upregulated and 11 were downregulated. Among the 11 *ClHSP20* genes upregulated by heat treatment, *ClHSP20-1, ClHSP20-2, ClHSP20-6, ClHSP20-16, ClHSP20-25, ClHSP20-26, ClHSP20-27, ClHSP20-30,* and *ClHSP20-31* increased dramatically to a peak (more than 100-fold) at 3 h and then started to decrease. On the other hand, the fold change of 11 downregulated *ClHSP20* genes (*ClHSP20-3, ClHSP20-4, ClHSP20-5, ClHSP20-9, ClHSP20-10, ClHSP20-11, ClHSP20-18, ClHSP20-19,* and *ClHSP20-32*) was below 100-fold.

The *HSP20* gene family not only plays a significant role in the response to heat stress but also participates in the response to drought stress. A total of 13 *ClHSP20* genes responded to drought stress at some or all of the time steps—3 h, 6 h, 12 h and 24 h—under 10% PEG6000 treatment, with 6 (*ClHSP20-1, ClHSP20-2, ClHSP20-20, ClHSP20-25, ClHSP20-28,* and *ClHSP20-32*) being significantly upregulated, and 5 (*ClHSP20-3, ClHSP20-9, ClHSP20-11, ClHSP20-18,* and *ClHSP20-19*) being significantly downregulated. Interestingly, *ClHSP20-16* was significantly upregulated at 3 h, significantly downregulated at 6 h and 12 h, and then significantly upregulated at 24 h. *ClHSP20-17* was significantly downregulated at 12 h and then significantly upregulated at 24 h under 10% PEG6000 treatment. The expression trends for most of the *ClHSP20* genes were similar under the heat and drought treatments, while some *ClHSP20* genes exhibited different responses under heat and drought stress. For example, 6 *ClHSP20* genes (*ClHSP20-6, ClHSP20-14, ClHSP20-26, ClHSP20-27, ClHSP20-30,* and *ClHSP20-31*) were induced, and 2 *ClHSP20* genes (*ClHSP20-10* and *ClHSP20-21*) were suppressed by heat stress but seemed to have no response to drought, while *ClHSP20-28* was induced by drought stress and seemed to have no response to heat stress. *ClHSP20-17* was suppressed at 12 h and induced at 24 h by drought stress and seemed to have no response to heat stress. In addition, the expression trends for most of the *ClHSP20* genes under the combined heat and drought treatment were consistent with those under the heat treatment (Fig. 7b; Supplementary Table S5). These results indicated that the *ClHSP20* genes were more sensitive under heat stress than under drought stress.

**Fig. 8:**
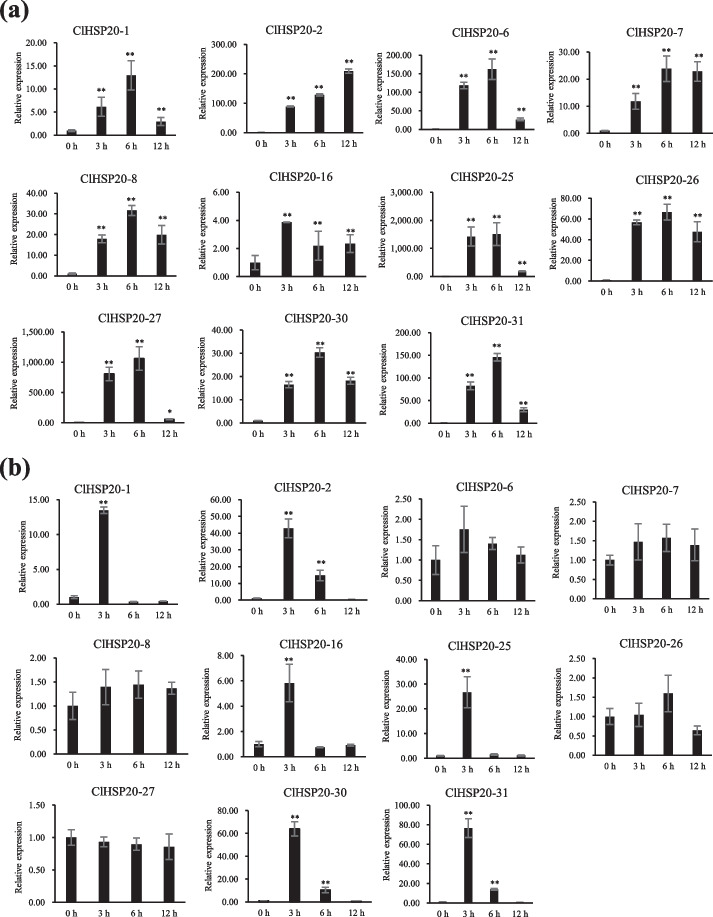
qRT–PCR analysis of 11 *ClHSP20* genes under heat and drought stress. **a** Expression pattern of *ClHSP20* genes under heat treatment (42 °C) for 0 h, 3 h, 6 h, and 12 h, respectively. **a** Expression pattern of *ClHSP20* genes under drought treatment (10% PEG6000) for 0 h, 3 h, 6 h, and 12 h, respectively. The column indicates were represented by mean ± standard deviation. All experiments were performed independently at least three times. Error bars represent the standard deviation of three replicates. ^*^ *P* < 0.05, ^**^P < 0.01

To validate the results obtained from the RNA-seq data, 11 *ClHSP20* genes were selected for further validation by qRT‒PCR (Fig. 8a, b). The expression trends of selected *ClHSP20* genes from the qRT‒PCR results were consistent with the RNA-seq data. It should be noted that *ClHSP20-6* and *ClHSP20-7* were dramatically upregulated after 3 h of heat treatment, but they showed no significant difference in the RNA-seq data because of the significant numerical difference between the three biological replicates, resulting in a large standard deviation. Furthermore, the expression profiles of 6 selected *ClHSP20* genes (*ClHSP20-1, ClHSP20-2, ClHSP20-16, ClHSP20-25, ClHSP20-30,* and *ClHSP20-31*) were upregulated at 3 h and then gradually decreased at 6 h and 12 h under 10% PEG6000 treatment, but 5 genes (*ClHP20-6*, *ClHP20-7*, *ClHP20-8*, *ClHP20-26*, and *ClHP20-27*) showed no significant differences under drought stress. The qRT‒PCR results generally confirmed the RNA-seq results. Overall, these results indicated that the *ClHSP20* genes may play critical roles in the response to heat and drought stress.

## Discussion

Numerous studies have shown that HSP20s are the most abundant family of *HSPs* involved as molecular chaperones in plant responses to abiotic stress [1, 6, 12]. Complete genome sequencing of Coix made it possible to analyse the *HSP20* gene family using genome-wide sequencing. In the current study, a total of 32 members of the *ClHSP20* gene family in Coix were identified and characterized for the first time (Table 1; Figs. 1 and 2). The number of *HSP20* genes in Coix was only greater than that in Arabidopsis (19) [21] and was less than that in rice (39) [7], tomato (42) [23], maize (44) [29], grape (48) [26], and soybean (51) [22] and far less than that in cotton (94) [24]. The genome size of Coix (1.2 G) is approximately 4.6 times that of Arabidopsis (260 Mb) [32], 3 times that of rice (389 Mb) [33], and half that of maize (B73, 2.106 G) [34]. However, the number of members of the *HSP20* gene family in these species is not proportional to their genome size.

Consistent with the *HSP20* gene family in other species, except as it pertains to the location of the endoplasmic reticulum, prediction of subcellular localization showed that 32 ClHSP20s were localized in the cytoplasm, nucleus, chloroplast, mitochondria, and peroxisome (Table 1). It should be noted that several Arabidopsis, rice, and maize HSP20 members are localized to the ER [29], but Coix lacks HSP20 members localized to the ER, indicating that the *HSP20* gene has gradually differentiated during the evolution process. Different subcellular locations of HSP20 proteins hint at the possibility that *HSP20* genes could play multiple functions in plants.

Phylogenetic analysis showed that 32 ClHSP20s were clustered into 10 subfamilies and an unclassified subfamily following HSP20 classification in Arabidopsis and rice (Fig. 1). Twenty-three of the 32 ClHSP20 (71.9%) were classified as CI–CVII, excluding CII, indicating that HSP20 plays an important role in the cytoplasm or nucleus. These results are similar to the findings in previous reports for other plant species. However, many plant species lack some of the HSP20 subfamilies; for example, Arabidopsis lacks the VI and CVII subfamilies [21], Populus lacks the CVI subfamily [35], rice lacks the CIV and CVII subfamilies [7], dove tree lacks the CII and CIII subfamilies [28], and pepper lacks the CIV and CV subfamilies [20]. Similarly, the ER, CIV, and CX subfamilies are absent from the *HSP20* gene family of Coix, indicating that gene loss and expansion are common phenomena during the evolution process. We found that the phylogenetic relationship between CP and MT is closer than that of other subfamilies, which is consistent with the findings of previous reports [17, 22, 25, 27]. Our results support the notion that the MT subfamily evolved later from the CP subfamily [1]. Interestingly, the expression profile of most *ClHSP20* genes sharply induced by heat stress belonged to the CI subfamily, while most *ClHSP20* genes suppressed by heat stress belonged to the P subfamily. The results suggested that the phylogenetic classification of *ClHSP20* genes was closely associated with their functions.

Gene duplication, including tandem duplication and segmental duplication, is the main driving force for the expansion of gene families and the evolution of the plant genome [36, 37]. The duplication and synteny analyses demonstrated that there were three tandem duplicated and three segmentally duplicated gene pairs in 32 *ClHSP20*s (Fig. 2, Table 2), indicating that tandem duplication and segmental duplication have promoted the expansion and evolution of the *ClHSP20* gene family. Similar duplication patterns were also found in rice [7], maize [29], and dove tree [28]. Interestingly, all three tandem duplicated gene pairs (*ClHSP20-10/ClHSP20-11; ClHSP20-25/ClHSP20-26; ClHSP20-30/ClHSP20-31*) and two segmentally duplicated gene pairs (*ClHSP20-14/ClHSP20-29; ClHSP20-22/ClHSP20-32*) displayed similar expression patterns in different tissues (Figs. 2 and 7a). In addition, all three tandem duplicated gene pairs (*ClHSP20-10/ClHSP20-11; ClHSP20-25/ClHSP20-26; ClHSP20-30/ClHSP20-31*) showed similar expression patterns under heat and drought stress (Figs. 2 and 7b). The results suggest that the biological functions of tandem and segmentally duplicated genes may be relatively conserved. On the other hand, the segmentally duplicated gene pairs *ClHSP20-2/ClHSP20-17* displayed different expression patterns both in different tissues and under heat stress (Figs. 2 and 7a, b), which hints at the biological functions of *ClHSP20-2* and *ClHSP20-17* having undergone differentiation during the evolution process.

**Table 2:** Ka/Ks value and divergent time of the duplicated *ClHSP20* gene pairs

Duplicated gene pairs	Type	Ka	Ks	Ka/Ks	Evolutionary pattern	Divergence Time (MYA)
*ClHSP20-10*/*ClHSP20-11*	Tandem	0.6319213	0.8230333	0.7677956	Purify selection	62.73
*ClHSP20-25*/*ClHSP20-26*	Tandem	0.0957111	0.2542068	0.3765089	Purify selection	19.38
*ClHSP20-30*/*ClHSP20-31*	Tandem	0.1245724	0.5038455	0.2472432	Purify selection	38.40
*ClHSP20-2*/*ClHSP20-17*	Segmental	0.4378276	0.6008867	0.7286358	Purify selection	45.80
*ClHSP20-14*/*ClHSP20-29*	Segmental	0.6698879	0.6000352	1.1164145	positive selection	45.73
*ClHSP20-22*/*ClHSP20-32*	Segmental	0.4372838	0.7181671	0.6088886	Purify selection	54.74

The structure of the exon‒intron plays a crucial role in the evolutionary and functional differentiation of the gene structure [38]. Analysis of the gene structure showed that most of the *ClHSP20* gene (87.5%) contained only one intron (62.5%) or no introns (25.0%) (Fig. 3), which aligns with the findings of previous studies on other *HSP20* gene families. Genes with fewer or no introns are considered conducive to being activated rapidly under stresses [39]. In this study, most *ClHSP20* of the CI subfamily did not have introns, whereas those of most of the other subfamilies had more than one intron. However, the gene structure of the *ClHSP20* genes in CI and CIII was different from that of grape [26], suggesting that the structure of the *ClHSP20* genes is different in some species. It should be noted that almost all of the *ClHSP20* genes sharply induced by heat and drought stress had no introns (Figs. 4 and 7b), which was in agreement with the findings of a previous report [39].

Further analysis of the evolution of ClHSP20s showed that almost all ClHSP20s contained motif 1 and motif 2, and that most ClHSP20s had 3–7 conserved motifs. Furthermore, the same ClHSP20 subfamily had similar motif structures, while the motif compositions were significantly different between different subfamilies, which indicated that the structure and function of *ClHSP20* were differentiated in the evolution process (Fig. 4c). Transcript expression analysis can help us to understand the potentially distinct functions of *ClHSP20s*. The transcriptional abundance of the 32 *ClHSP20* genes varied greatly in different vegetative organs and reproductive organs, suggesting their redundancy and diverse function in growth and development (Fig. 7a). Some *ClHSP20* genes, especially these six pairs of tandem and segment duplicated genes, showed the most similar expression patterns, hinting that these *ClHSP20s* may have potential redundant functions in growth and development. Several *ClHSP20* genes, including *ClHSP20-3*, *ClHSP20-4*, *ClHSP20-20*, and *ClHSP20-21*, were highly and universally expressed in all tissues detected under normal conditions. Similar *HSP20* housekeeping genes were also found in soybeans [22] and potato [25]. Several *ClHSP20* genes showed tissue-specific expression patterns, such as preferential expression in reproductive organs (kernel, glume, and male flower) or high expression in certain tissues (*ClHSP20-18* in leaf, *ClHSP20-5* in root, *ClHSP20-17* in male flower, *ClHSP20-23* in shoot, *ClHSP20-6*, and *ClHSP20-30* in kernel) (Fig. 7b), hinting that they probably play different roles in certain tissues. Previous reports have demonstrated that some *HSP20* genes are involved in plant growth and development processes, including hypocotyl elongation, pollen development, seed maturation, and germination [1]. For example, *AtHSP22* plays an important role in auxin-regulated hypocotyl elongation at high temperatures in Arabidopsis [40]. *PpHSP20-32* was found to participate in the development of plant height in peach [17]. The *sHSP*s in sweet pepper were demonstrated to play important roles in fruit ripening associated with the process of physiological nitro-oxidative stress [31].

The GO enrichment analysis showed that the highly enriched GO terms included response to heat (GO:0009408) in the biological process terms and included *ClHSP20-1*, *ClHSP20-2*, *ClHSP20-6*, *ClHSP20-7*, *ClHSP20-8*, *ClHSP20-16*, *ClHSP20-17*, *ClHSP20-18*, *ClHSP20-25*, *ClHSP20-27*, *ClHSP20-28*, and *ClHSP20-30*. According to the RNA-seq and qRT‒PCR data, the expression levels of most of these *ClHSP20* genes, except for those of *ClHSP20-17*, *ClHSP20-18*, and *ClHSP20-28*, were sharply increased under heat stress. Interestingly, *ClHSP20-18* was significantly decreased under heat stress, which also aligns with the results of the GO enrichment analysis (Fig. 5a).

The promoter regions of the *ClHSP20* genes were predicted to harbour multiple stress-responsive, hormone-responsive, and plant development-related *cis*-acting elements (Fig. 6), suggesting that the *ClHSP20* genes could have distinct functions. GO enrichment analysis showed that the *ClHSP20* genes were highly enriched in response to abiotic, heat and temperature stimuli. The KEGG enrichment analysis showed that the *ClHSP20* genes were highly enriched in the protein folding, sorting and degradation pathway and the folding catalyst pathway (Fig. 5; Supplementary Table S2 and S3). The expression levels of eleven *ClHSP20* genes sharply increased to a peak at 3 h under heat stress, suggesting that these *ClHSP20* genes might play a critical role in the response to heat stress. In addition, most of the *ClHSP20*s responded to drought stress, and their expression trends were completely similar to those under heat stress. Furthermore, the expression profiles of most of the *ClHSP20* genes under heat stress were generally similar to those under heat stress combined with drought stress (Supplementary Table S5; Fig. 7b). The results agreed with those of most studies on *HSP20s* in rice [7], pepper [20], maize [29], and enhanced rice [29]. The results suggested that interactions occurred in these *ClHSP20* genes in response to different abiotic stresses, and that *ClHSP20* genes were more sensitive to heat stress than to drought stress. In general, our study provides new comprehensive information and will aid in further functional characterization of *HSP20* genes in Coix.

## Conclusions

In summary, a total of the 32 ClHSP20 members were identified for the first time from the genome of Coix in the current study. Gene duplication analysis showed that tandem and segmental duplication had promoted the expansion of the *ClHSP20* gene family. Almost all six duplicated *ClHSP0* gene pairs, except *ClHSP20-14/ClHSP20-29*, underwent purification selective pressure during evolution. Many orthologous *ClHSP20* gene pairs were identified between Coix and other species, indicating high synteny among Coix and cereal plants. The GO, KEGG, and PPI analyses demonstrated that the biological functions of *ClHSP20* genes are involved in the development and response to abiotic stresses. The expression patterns of the *ClHSP20* genes suggested that they play a critical role in growth and development, as well as in response to heat and drought stress. Overall, the present study provides a theoretical basis for further research on *ClHSP20s* and will facilitate the functional characterization of *ClHSP20* genes.

## Materials and methods

### Identification and chromosomal location of *ClHSP20* genes in Coix

The whole genome file was downloaded from the BIG Data Center genome database (https://ngdc.cncb.ac.cn/, accession number: GWHAAYR00000000), and a database of nonredundant Coix proteins (*C. lacryma-jobi* L) was constructed [39]. Two methods were performed to screen candidate *HSP20* genes: 1) the hidden Markov model (HMM) of the conserved domain of HSP20 (PF00011) was obtained from the Pfam database (http://pfam.xfam.org/, accessed on 1 March 2023) and was used to search the database of nonredundant Coix proteins using the HMMER3 program with default parameters. 2) Arabidopsis HSP20 protein sequences (downloaded from the TAIR database (https://www.arabidopsis.org/, accessed on 1 March 2023) and Rice Genome Annotation Project database (http://rice.uga.edu, accessed on 1 March 2020) were used as queries to search the Coix nonredundant protein database using the BLASTp program (E-value < 1e-5). To confirm the identification of the *ClHSP20* gene family members, all candidate ClHSP20 protein sequences were removed from redundant sequences to retain unique *HSP20* genes. Then, the unique HSP20s were further verified in the NCBI-CDD (https://www.ncbi.nlm.nih.gov/cdd, accessed on 2 March 2023), Pfam (https://pfam-legacy.xfam.org, accessed on 1 March 2023), and SMART (http://smart.embl.de/, accessed on 2 March 2023) databases.

Information on the chromosome location, protein, and CDS length of *ClHSP20* was obtained along with the Coix genome. MG2C (MapGene2Chrom, http://mg2c.iask.in/mg2c_v2.0/, accessed on 3 March 2023) was used to map the chromosomal position of the *ClHSP20* genes. Furthermore, the physicochemical properties of the ClHSP20 proteins were predicted in Expasy (https://web.expasy.org/compute_pi/, accessed on 3 March 2023). The subcellular location of ClHSP20 was predicted by the online analysis tools Euk-mPLoc 2.0 (http://www.csbio.sjtu.edu.cn/bioinf/euk-multi-2/, accessed on 3 March 2023) and WoLF_PSORT (https://wolfpsort.hgc.jp/, accessed on 3 March 2023).

### Gene duplication and collinearity analysis

The *HSP20* gene duplication in Coix, rice, sorghum, and maize was performed by MCScanX with the default parameters. Collinearity analysis between Coix and seven other plant species was performed using TBtools software (v1.098774) with the one-step MCScanx command. The synonymous substitution rates (Ks), nonsynonymous substitution rates (Ka), and Ka/Ka ratio of *HSP20* gene pairs were calculated by KaKs_Calculator 2.0 software. The divergence time (T) was calculated using the Formula *T* = Ks/2r × 10 − 6 Mya (*r* = 6.56 × 10^−9^ for grasses) [41].

### Phylogenetic analysis, gene structure, and motif analysis

For phylogenetic analysis, the protein sequences of the Coix, Arabidopsis, and rice *HSP20* gene families were aligned with Clustal-X 1.83 with default parameters [42]. The phylogenetic tree was constructed using MEGA 7.0 with the neighbour-joining (NJ) method with 1,000 bootstrap replications [43]. The detailed parameters are as follows: the alignment sequences selected = MUSCLE method; Gap open and Gap extend = 2.9 and 0, respectively; the Poisson model = substitution model; uniform rates = gaps/missing data = pairwise deletion, respectively, with other values kept to the default. The phylogenetic tree was visualized via Evolview (http://www.evolgenius.info/evolview, accessed on 20 May 2023).

The conserved domain of ClHSP20s was predicted using the NCBI-CDD (https://www.ncbi.nlm.nih.gov/cdd). The conserved motifs of the ClHSP20s were identified with MEME (https://meme-suite.org/meme/tools/meme) (accessed on 5 March 2023) with the following parameters: the optimum motif width was 6 to 200, and the maximum number of motifs was 15. The obtained CDD domain and the conserved motifs of the ClHSP20 protein, together with intron‒exon structures of the *ClHSP20* genes, were visualized using Tbtools software (v1.098774) [44].

### Functional analysis of ClHSP20

Gene Ontology (GO) and Kyoto Encyclopedia of Genes and Genomes (KEGG) (www.kegg.jp/kegg/kegg1.html) annotation evaluations were conducted by submitting the ClHSP20 protein sequences to eggNOG-mapper [45, 46]. Then, GO and KEGG enrichment evaluations and visualizations were performed using TBtools. The protein‒protein interaction (PPI) network of the ClHSP20 proteins was generated using the STRING database V11.5 (https://cn.string-db.org/, accessed on 6 March 2023).

### Analysis of *cis*-acting elements in* ClHSP20* promoters

The 2,000 bp upstream of the transcription initiation site ATG of *ClHSP20s* was obtained and predicted with the online PlantCARE database (https://bioinformatics.psb.ugent.be/webtools/plantcare/html/, accessed on 6 March 2023).

### Expression pattern of *ClHSP20* genes in different tissues and response to heat and drought stress

The transcriptomic data were downloaded from the NCBI public database (BioProject number: PRJNA544168 and PRJNA812268). The average TPM value of each repetition was converted to log2 and visualized using the heatmap of TBtools software (v1.098774) [44].

### Plant growth, abiotic stress and hormone treatment, tissue collection

The Coix were grown in plastic pots with sand in a greenhouse under 25 °C-28 °C temperature, 75% humidity, and 14 h light/10 h darkness photoperiod. Two-week-old seedlings were subjected to heat stress (42 °C) for 0, 3, 6, and 12 h. The leaf samples were instantly frozen in liquid nitrogen and stored at − 80 °C. Each treatment included six pots of seedlings with a uniform growth trend. Three biological replicates were collected for each sample.

### Validation of *ClHSP20* gene expression by qRT‒PCR

Total RNA was extracted by TRIzol reagent (Invitrogen, Beijing, USA), and cDNA was synthesized using the AT311-03 cDNA kit (TransGen Biotech, Beijing, China) according to the manufacturer’s instructions. qRT‒PCR was carried out using THUNDERBIRD qPCR Mix QPS-201 (Toyobo, Shanghai, China) and an ABI 7500 Real-Time PCR System (Applied Biosystems, Waltham, CA, USA). The PCR program was as follows: 10 min at 95 °C, with 40 cycles of 15 s at 95 °C and 60 s at 55 °C. Statistical analysis was performed after obtaining the Ct values from three biological replicates. The *UBQ5* gene was employed as the internal reference. The ∆∆Ct values were calculated and presented as the means ± standard errors (SE) of three replicates. The PCR primers utilized in this study are listed in Supplementary Table S6.

### Supplementary Information

**Additional file 1: Supplementary Table S1. **GO Enrichment of ClHSP20s.

**Additional file 2: Supplementary Table S2. **KEGG Enrichment of ClHSP20s.

**Additional file 3: Supplemental Table S3. **Critical cis-regulatory elements distributed in the promoter of *ClHSP20* genes.

**Additional file 4: Supplemental Table S4. **The TPM values for different tissues of the *ClHSP20* genes base on RNA-seq.

**Additional file 5: Supplemental Table S5. **The TPM valuesof the *ClHSP20* genes under heat and drought tratment based on RNA-seq data.

**Additional file 6: Supplemental Table S6. **The primers of selected *ClHSP20* genes for qRT-PCR.

## Data Availability

The datasets supporting the results of this article are included within the manuscript and available on request (Dr. Dekai Wang). The Coix (*Coix lacryma-jobi*) genome DNA sequencing data have been deposited into BIG Data Center (https://ngdc.cncb.ac.cn/, accession number: GWHAAYR00000000). The transcriptomic data were downloaded from the NCBI public database (BioProject number: PRJNA544168 and PRJNA812268).
