# Supplementary material for: Genome-wide analysis of the HSP20 gene family and its response to heat and drought stress in Coix (Coix lacryma-jobi L.)
Source: BMC Genomics. 2023 Aug 24;24:478. doi: 10.1186/s12864-023-09580-2 (PMC10464217; doi:10.1186/s12864-023-09580-2)
Supplement: Supplementary file 6 — Additional file 6: Supplemental Table S6. The primers of selected ClHSP20 genes for qRT-PCR. [file 12864_2023_9580_MOESM6_ESM.docx]

| **Primer** | **Sequence (5’-3’)** |
| --- | --- |
| ClHSP20-1F | GACGCTGGAGGAGGAGGA |
| ClHSP20-1R | CTCCAGCCGGATGTAGCG |
| ClHSP20-2F | CGCCTCCGAGATACAGCG |
| ClHSP20-2R | ACGACCACCTTGAGCACG |
| ClHSP20-6F | TGCAGGTGAGCCAGAACG |
| ClHSP20-6R | ACCGTGTCCAGCATCTGC |
| ClHSP20-7F | TGTTCAAGGCGGACGTCC |
| ClHSP20-7R | CTCGCCGCTGATCTGGAG |
| ClHSP20-8F | TGTTCAAGGCGGACGTCC |
| ClHSP20-8R | CTCGCCGCTGATCTGGAG |
| ClHSP20-16F | CGTGCTCACCATCAGGGG |
| ClHSP20-16R | TCCACACGCACGTTCTCC |
| ClHSP20-25F | CCTCTGGGACCCCTTCGA |
| ClHSP20-25R | AGGTCGGCCTTGAACACG |
| ClHSP20-26F | GAGGTGGAGGACGGCAAC |
| ClHSP20-26R | TGGTCCGTCTTGGCGTTC |
| ClHSP20-27F | CCTCTGGGACCCCTTCGA |
| ClHSP20-27R | AGGTCGGCCTTGAACACG |
| ClHSP20-30F | TGTTCAAGGCCGACGTCC |
| ClHSP20-30R | CTCGCCGCTGATCTGGAG |
| ClHSP20-31F | GCAACAGGGAGCAGGAGG |
| ClHSP20-31R | GAGCACGCCGTTCTCCAT |
| UBQ5-F | TGCTGCAGTTCTACAAGGTG |
| UBQ5-R | GAAGTGGTTGGCCATGAAG |

Supplemental Table S6. **The primers of selected *ClHSP20* genes for qRT-PCR**
